# Does Flooring Substrate Impact Kennel and Dog Cleanliness in Commercial Breeding Facilities?

**DOI:** 10.3390/ani8040059

**Published:** 2018-04-21

**Authors:** Judith Stella, Moriah Hurt, Amy Bauer, Paulo Gomes, Audrey Ruple, Alan Beck, Candace Croney

**Affiliations:** 1 United States Department of Agriculture, Animal and Plant Health Inspection Service, 625 Harrison St, West Lafayette, IN 47907, USA; judith.l.stella@aphis.usda.gov; 2 Department of Comparative Pathobiology, Purdue University, 625 Harrison St, West Lafayette, IN 47907, USA; mhurt2096@gmail.com (M.H.); aebauer4@gmail.com (Am.B.); aruplecz@purdue.edu (A.R.); abeck@purdue.edu (Al.B.); 3 Department of Veterinary Clinical Sciences, Purdue University, 625 Harrison St, West Lafayette, IN 47907, USA; gomesp@purdue.edu

**Keywords:** dogs, flooring, health, well-being, cleanliness

## Abstract

**Simple Summary:**

It is important to understand how the flooring substrate used in dog housing impacts dog health and well-being. Aspects to consider include paw, elbow, and hock health, the cleanliness of the dog, and the ability of the floors to be cleaned easily and thoroughly. This pilot study assessed the health and cleanliness of 118 dogs housed on three different types of flooring commonly found in commercial breeding kennels. No serious paw, elbow, or hock problems were identified. Thirty-one percent or fewer kennels at each facility were found to have fecal contamination after routine cleaning and the majority of dogs were clean. These findings indicate that a well-managed kennel can maintain clean, healthy dogs on different types of flooring substrates.

**Abstract:**

Evaluation of kennel flooring surfaces is needed to understand their impacts on dog health and well-being. This pilot study aimed to characterize aspects of physical health, kennel cleanliness, and dog body cleanliness on flooring types common in US breeding kennels. Subjects were 118 adult dogs housed on diamond-coated expanded metal (DCEM), polypropylene (POLY), or concrete (CON) flooring at five commercial breeding facilities in Indiana, U.S. Body condition, paw, elbow, and hock health scores were recorded. Each indoor kennel and dog was visually assessed for cleanliness. Kennels were swabbed immediately after cleaning with electrostatic dry cloths and cultured for *Escherichia coli*. Descriptive statistics were used for analysis. Mean body condition score (BCS), kennel and dog cleanliness scores were all near ideal (3, 1.15, and 1.04, respectively). Thirty-one percent or fewer kennels at each facility were culture-positive for *E. coli* after cleaning. No serious paw, elbow, or hock problems were identified. Overall, the findings indicate that with appropriate management and regular access to additional surfaces, dog foot health, cleanliness, and kennel cleanliness can be maintained on the flooring types investigated.

## 1. Introduction

The high demand for purebred dogs that exists in the U.S. is partially met by the commercial dog breeding industry. However, many concerns exist about the welfare of dogs raised in commercial or high-volume breeding kennels, prompting proposed regulatory changes to breeder practices. The paucity of research conducted in commercial breeding (CB) facilities to date raises questions about the scientific basis and soundness of several of the recommendations, as the welfare effects of many aspects of dog housing and management are unknown. One area that is of particular concern in CB kennels is the type of flooring used and its implications for dog well-being. While research has been conducted on topics such as the quality and quantity of space provided to dogs and the effects of environmental enrichment in kennel environments [1,2,3,4,5,6,7,8,9], little research exists on impacts of different aspects of housing, such as flooring [10], on dog welfare and no published studies appear to have investigated these areas in commercial breeding operations.

Although little has been published on the effects of flooring on dog well-being, research in other species has demonstrated that inappropriate flooring may negatively impact foot health. For example, Haspeslagh et al. [11] reported that elephants housed on sand and concrete were more likely to have foot health problems than those housed on other substrates, while turkeys housed on damp litter have been shown to develop more foot ulcers than birds housed on dry litter [12]. Effects of flooring on dairy cow well-being are also well established. Cows maintained on rubber slats appeared less likely to experience foot disease than those housed on solid rubber flooring. Further, those housed on concrete were more prone to develop lameness, and heel erosions than those kept on solid rubber [13], and rubberized alley flooring has been suggested to reduce dairy cow claw wear and trauma [14].

In addition, flooring can significantly impact animal welfare by promoting or undermining animal comfort, safety, and cleanliness. For example, Brscic et al. [15] suggested that beef cattle housed on rubber slatted flooring were more comfortable than those kept on concrete slats, resulting in more time spent standing and eating and, consequently, higher average daily gain in those maintained on rubber flooring. Likewise, dairy cows housed with access to geotextile mattresses and those kept on rubber matted floors spent more time standing while not eating than those maintained on concrete [16]. Different flooring surfaces may also impact animal gait and ability to move safely and without slipping [17]. For instance, dairy cows display different stride lengths on solid rubber versus slatted rubber flooring, indicating that they attempt to change or shorten their stride to avoid slipping [18].

Finally, the impact of flooring on maintaining animal cleanliness must be considered. Beef cattle housed on solid flooring were found to be dirtier than those housed on slatted flooring [19] and farmed foxes housed in cages with access to sand had dirtier coats during pelting compared to foxes housed on mesh flooring [20]. Presumably, these effects are due at least in part to the open flooring permitting waste to fall below the pen and away from the animals. However, the use of non-solid flooring for dog housing is a highly contentious issue. In an attempt to comply with existing regulations, commercial dog breeders and others operating kennels may elect to use slatted or other non-solid flooring in an effort to improve dog cleanliness and maintain generally sanitary conditions. In fact, the ability to remove waste from the kennel on a daily basis is required [21]. Further, the flooring surfaces must be easy to sanitize and drain of excess water. Visual cleanliness of the kennel can be improved by daily cleaning, but pathogen load is impacted by characteristics of the flooring type [22] as well as the cleaning protocol and the effectiveness and frequency of the disinfectants used. Ensuring that kennels are clean of microbial contamination in addition to being visibly clean is also important to minimize health risks to dogs.

With these considerations in mind, the use of non-solid flooring may appear well justified to many kennel managers. However, several factors relative to flooring choice for dogs in breeding facilities raise concern. These include the use of wire strand flooring and improperly sized slats or openings. The short- and long-term effects of dogs’ inability to routinely traverse multiple flooring substrates, particularly when only non-solid flooring is used, must also be considered. Paw, hock, and/or elbow damage are possible outcomes. Additionally, for dogs intended to be rehomed at the end of their breeding careers, behavioral problems may occur, such as inappropriate elimination if the substrates dogs have learned to eliminate on are then unavailable in homes. This may create indirect dog welfare problems, such as owner dissatisfaction with, and ultimately, surrender of dogs [23]. These concerns have led to calls to regulate breeders and limit the flooring choices they make. For example, recent legislation in Missouri banned the use of wire-strand flooring in all facilities subjected to regulation under the Canine Cruelty Prevention Act of 2011, effective 2016.

Because relatively little is known about how specific flooring types may impact foot health and other aspects of dog well-being, it is important to evaluate these factors. The social contention around high volume dog breeding and the need for informed policy development relative to appropriate housing of breeding dogs make investigation of this population necessary. We therefore aimed to begin a line of inquiry into the welfare implications of housing dogs on different flooring types in commercial breeding (CB) facilities. Given publicly reported concerns about the safety and physical health of dogs in CB kennels and associated regulatory focus on the flooring types on which they are kept, the pilot study reported here had several goals. We sought to understand the current state of foot health in dogs maintained in commercial breeding (CB) facilities in the United States, visually examine the cleanliness of the dogs and their kennel floors, and evaluate the level of fecal contamination after routine cleaning of diamond-shaped coated expanded metal (DCEM), polypropylene (POLY), and concrete (CON) flooring types.

## 2. Materials and Methods

### 2.1. Subjects and Facilities 

**Figure 1:**
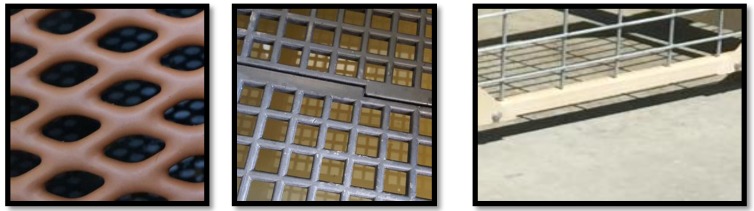
Flooring types. Indoor flooring assessed from far left: diamond-shaped coated expanded metal (DCEM), polypropylene (POLY), and concrete (CON).

Five Amish-owned CB kennels volunteered for participation in the study. Data were collected via scheduled visits during regular business hours (09:00–17:00). A random sample of healthy intact adult dogs (n = 118, 95 F, 23 M) over one year of age (mean 37.9 months, range 12–118 months), representing 13 breeds were assessed on-site at their home facilities (Table 1). Bitches in the last two weeks of gestation or those nursing puppies were excluded as was any dog that exhibited overt fear or distress prior to or during physical exam. Dogs were single, pair, or group housed in a kennel that consisted of an indoor run with access to an outdoor concrete run and turn out space in an exercise yard that was either grass, gravel, or a combination of the two. The flooring substrates assessed in this study were the indoor surfaces in use at each facility. These included diamond-shaped coated expanded metal (DCEM), polypropylene (POLY), or sealed concrete (CON) (Figure 1, Table 2). Informed consent was obtained from all facility owners who volunteered for the study. The Institutional Animal Care and Use Committee, Institutional Review Board, and the Clinical Trials Office in the College of Veterinary Medicine at Purdue University approved all experimental procedures.

### 2.2. Metrics

A licensed veterinarian conducted all physical examinations to assess dogs’ paws, elbows, hocks, and body conditions. The cleanliness of dogs’ bodies and their respective kennels were visually scored using a scale developed for the research and recorded at the time of the physical examination.

### 2.3. Body Condition Score (BCS) 

**Figure A1:**
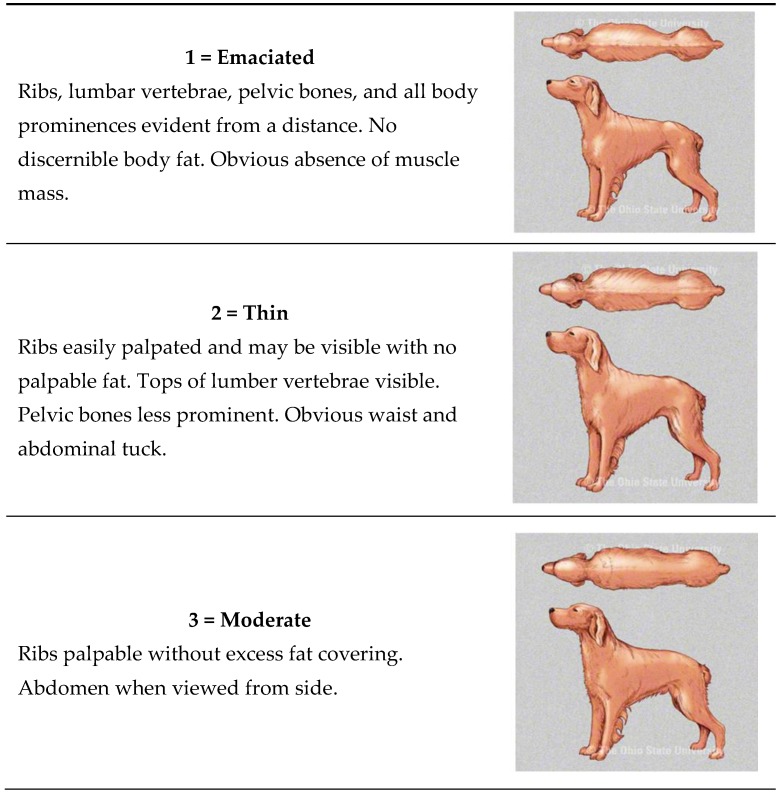

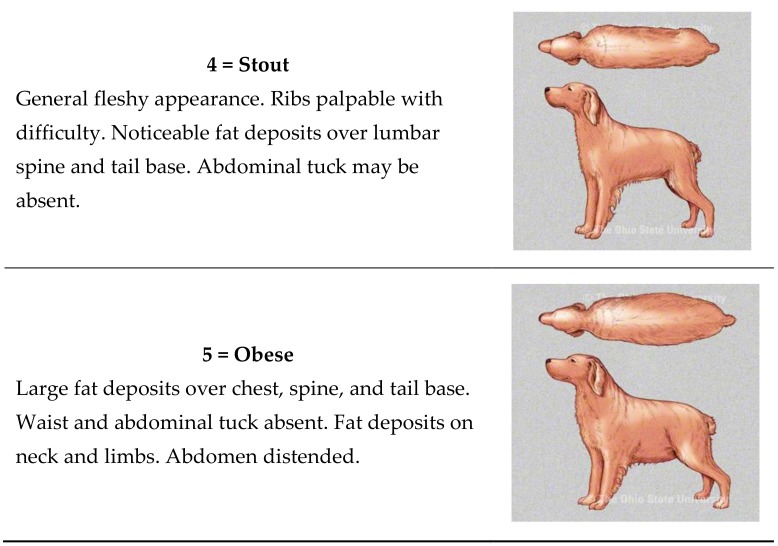
Body condition scoring. The tool used to assess BCS (https://vet.osu.edu/vmc/).

Body condition of dogs was collected to determine its possible relationship to the development of adverse foot health conditions in dogs housed on different flooring substrates. Body condition was scored using a five-point scale (1 = emaciated; 3 = ideal; and 5 = obese) from The Ohio State University’s Veterinary Medical Center (https://vet.osu.edu/vmc/) (Figure A1).

### 2.4. Elbow and Hock Health 

Both elbows and hocks were assessed for the presence of any abnormalities (Table 3).

### 2.5. Paw Health 

Each paw was assessed, including interdigital areas, the pads, and the toenails, for evidence of the presence of any abnormalities (Table 3). Toenail length was scored for each individual toenail using a five-point scale developed by the experimenters as follows: 1 = normal length; 2 = slightly overgrown; 3 = overgrown but not curling; 4 = beginning to curl, ± growing into the pads, ± broken or missing nails; and 5 = curling, growing into the pad, cracked and/or missing nails. Any abnormalities of the toenails (e.g., onycholysis, onychoschizia, onychorrhexis, onychodystrophy) were also recorded. An average score for each foot was calculated.

### 2.6. Visual Kennel Cleanliness Scores (KCLS) 

Visual kennel cleanliness scores (KCLS) for the indoor portion of the home kennel (floor and walls) were recorded when the dog was removed for the physical exam. KCLS were based on a five-point scale as follows: 1 = no debris present in the kennel; 2 = 1–25% of the kennel covered in debris; 3 = 26–50% of the kennel covered in debris; 4 = 51–75% of the kennel covered in debris; and 5 = >75% of the kennel covered in debris (Table A1). Kennel cleaning occurred between 04:30–05:30 each morning; KCLS were collected between 09:00–17:00.

### 2.7. Body Cleanliness Score (BCLS) 

Body cleanliness scores (BCLS) were recorded based on a five-point scale developed by the experimenters as follows: 1 = no debris present on the dog; 2 = debris present on the paws only (1–25% of dog’s body); 3 = debris present on the legs, chest, and/or abdomen (26–50%); 4 = debris covering most of the dog except his/her head and neck (51–75%); and 5 = debris present on all parts of the dog (>75%) (Table A1).

### 2.8. Environmental Swabs 

Kennel flooring surfaces were swabbed and cultured for the presence of *E. coli*, a species commonly used to indicate fecal contamination. A random sample of 111 indoor kennel floors were swabbed after daily routine cleaning procedures, which entailed hosing the kennel floor and walls with soap and water. The entire kennel floor was swabbed using Swiffer electrostatic dust cloths (Proctor and Gamble, Cincinnati, OH, USA), following previously published techniques [24,25,26,27,28]. The cloth was removed with gloves and placed into a sterile Whirl-Pak (Nasco, Fort Atkinson, WI, USA). The Swiffer mop heads were sprayed with 70% ethanol and allowed to air dry between samples.

Samples were stored in a conventional refrigerator at approximately 1.6 °C until submission to the Animal Disease Diagnostic Laboratory at Purdue University for culture of *E. coli*. Culture proceeded as follows using aseptic techniques: 100 mL of phosphate buffered saline (PBS, pH 7.4) was added to the Whirl-Pak containing the electrostatic cloth. The PBS and cloth were mixed together inside the closed Whirl-Pak and allowed to sit for 10 min to permit the PBS to soak throughout the cloth. While still in the Whirl-Pak, the cloth was squeezed to expel the PBS from the cloth and 100 μL of the resulting liquid was removed from the Whirl-Pak and plated on a MacConkey agar plate using an “L” shaped spreader for confluent growth. The MacConkey agar plate was left undisturbed for 10 min to allow the agar to absorb the liquid. Plates were then inverted and incubated at 37 °C (±2 °C) for 18–24 h. Plates were then examined using standard protocols for identifying *E. coli* (i.e., growth of pink to red colonies with a bile salt precipitate surrounding the colonies). *E. coli* was further identified using the Matrix-assisted laser desorption/ionization mass spectroscopy (MALDI) using Bruker Daltonik MALDI Biotyper version 3.1 identification software (model MTX-LRF™ by Bruker Corporation, Billerica, MA, USA) under standard procedures.

### 2.9. Statistical Analysis 

Descriptive statistics (e.g., means, standard deviations) were used within facilities since variation in breed and management prohibited direct comparisons across facilities and further generalizing of results. Analysis was performed using STATA IC 11 (StataCorp LP, College Station, TX, USA) statistical software.

## 3. Results

A summary of the findings of the physical exams is presented in Table 4.

### 3.1. Body Condition

The mean body condition score for all dogs assessed was 3.2, ranging from 2.0 (n = 5) to 5.0 (n = 4).

### 3.2. Elbow, Hock, and Paw Health

The majority of abnormalities of the elbows and hocks were alopecia (n = 15) and calluses (n = 3). The majority of paw conditions identified were matted fur between the paw pads (n = 48), mild inflammation (n = 12), sores (n = 2), and two cases of interdigital furuncles (cysts) were identified.

### 3.3. Body Cleanliness

Dogs at all facilities appeared to be quite clean (mean score 1.04 ± 0.2, range: 1–2) (Table 5).

### 3.4. Visual Kennel Cleanliness

Kennels at all facilities appeared to be clean 4–12 h post cleaning (mean score 1.12 ± 0.36, range: 1–2) (Table 6).

### 3.5. Environmental Swabs

The percentage of kennel floors that cultured positive for *E. coli* ranged from a low of 7% at facility 3 to a high of 31% at facility 1 (Table 7).

## 4. Discussion

This pilot study focused on evaluating the physical health and cleanliness of dogs, and the level of hygiene achieved as a function of the flooring type used in the commercial breeding kennels in which they were maintained. The study has several important findings. First, in contrast to widespread reported concerns [29], the dogs at the commercial breeding kennels investigated appeared to be clean, in good body condition, and have few foot health problems. While it was difficult to generate and test hypotheses given both the lack of previous investigation with this population of dogs, and the nature of the study design, it was anticipated that there would be paw health problems on some of the floors, that these would be more severe on non-solid floors, and that there would be variation in body condition scores that might permit correlation between these factors. However, the finding of few significant elbow, hock, or paw health problems along with the majority of dogs having body condition scores (BCS) in the ideal range made it impossible to determine whether body condition in combination with any of the flooring types was associated with increased risk for paw abnormalities. An earlier study, aimed at identifying factors associated with the development of interdigital cysts in laboratory beagles housed on different flooring types, reported that dogs with higher BCS housed on a flat-bar flooring substrate had more interdigital cysts than those with lower BCS [10]. Further research is needed with breeding dogs with more variation in BCS to determine if indeed a relationship exists between body condition, flooring type, and adverse paw health outcomes.

In regard to elbow and hock problems, alopecia and calluses were most commonly identified. These were observed mainly in the large breed Rottweiler and Weimaraner dogs. Alopecia and calluses on the elbows of large breed dogs may occur especially when they have increased contact with the ground [30]. The fact that these problems were mostly found on the hocks and not the elbows is unusual and may be related to the dogs’ resting position in the kennel. One possible explanation is that bitches in late pregnancy or those nursing pups may not spend much time in sternal recumbency, reducing the amount of contact elbows have with abrasive flooring. These dogs may have spent more time in lateral recumbency or in a sitting posture when resting, reducing pressure on the elbows. Given that the larger breed dogs were more commonly housed on concrete, a more abrasive flooring surface than DCEM or POLY, it is possible that this may have resulted in more instances of alopecia and calluses. Because Rottweilers and Weimaraners are short-haired breeds, they may also have less protection around elbows and hocks and thus may be more prone to developing alopecia and calluses in these areas than long-haired breeds. Further research is needed to determine the postures adopted by resting dogs, including those at different stages of gestation, and the influence these may have on the development of inflammation, alopecia, and calluses on elbows and hocks.

Most other paw health problems observed were mild and unlikely to significantly impact dog welfare, such as matting of fur of the interdigital or palmoplantar regions. Breed differences were observed here, with long-haired breeds (Yorkshire Terrier, Maltese, Wheaton Terrier) having more matted fur on their paws. This finding suggests a need for more frequent grooming of the paw hair, particularly in long-haired dog breeds. Matted hair of the interdigital or palmoplantar regions of the paw can trap moisture and debris close to the skin, which can potentially alter the skin pH. Because this is believed to impact normal keratinization, desquamation, permeability, and the antimicrobial properties of the skin [31,32], routine trimming of paw hair as a means of maintaining good foot health in dogs is important.

The dogs assessed in this study had healthy toenails of appropriate length, indicating that even when the indoor kennel flooring types were not abrasive enough to allow natural grinding of toenails, regular trimming was effective at keeping nails in good condition. Rear toenails were slightly shorter than front toenails, probably because dogs place more pressure on their rear feet when they begin running [33].

Finally, all dogs and the majority of kennels were clean based on both visual assessments and pathogen swabs, indicating that despite differences in frequency (e.g., once or twice a day) and cleaning protocols (e.g., scrubbing with a brush or hosing only) across facilities, reasonably hygienic conditions were attained. The use of flooring types that were easy to clean in combination with daily removal of debris likely led to decreased time the dogs spent in close proximity to excrement, resulting in the relatively low level of fecal contamination found in most kennels sampled and the unsullied appearance of the dogs and kennels observed. These findings are similar to those of other studies reporting greater cleanliness in animals housed on non-solid flooring. Farmed foxes had cleaner pelts when housed solely on wire mesh flooring compared to foxes with access to sand flooring [20]. Similarly, beef cattle were cleaner when housed on slatted flooring surfaces compared to solid rubber flooring [21]. In both studies, the authors suggested that solid surfaces (i.e., sand and solid rubber) became dirtier with time and increased the animals’ exposure to urine and feces due to poor drainage of excrement.

Fecal coliforms were identified in an average of 23.7% of samples taken from kennel floors after routine cleaning, which indicates that cleaning procedures at these facilities may be ineffective at removing all fecal contaminants. Alternatively, the sampling technique may not have been effective at capturing *E. coli*. However, previous studies using Swiffer electrostatic dry cloths to collect *E. coli* and *Salmonella* spp. have not reported problems with the technique [22,23,27,28]. Another explanation is that use of high pressure water sprayers to remove fecal contamination in adjacent dirty kennels could aerosolize *E. coli*, leading to kennel contamination after cleaning procedures have occurred. Nonetheless, since samples were taken after routine cleaning rather than disinfection, some positive samples were expected. It should also be noted that only presence or absence of *E. coli* was noted; coliform counts would be needed to more precisely characterize the level of fecal contamination in kennels after routine cleaning. Without these, it is not possible to determine whether only a few or many coliforms were present in the culture positive samples, which would in turn better inform our understanding of consequent health and hygiene risks to dogs. Overall, the fact that there were contaminated kennels after cleaning in each facility suggests a need for standardized, effective cleaning protocols in CB kennels and the establishment of reference ranges for coliform recovery relative to promoting best dog health and hygiene outcomes

It is important to note that this study has several limitations. The small number of facilities from the same geographical location as well as the small number of flooring types assessed limit the generalizability of this study. Future studies should aim to expand the generalizability of these findings and to assess the preferences of dogs for different flooring substrates, as outstanding questions remain about the behavior and comfort of dogs on different flooring types and answers are needed to fully understand their welfare impacts. Further, to better understand the potential protective impacts of dogs experiencing additional outdoor surfaces, future studies should aim to compare foot health in dogs maintained solely on single flooring types with matched control animals allowed access to other surfaces. Investigation is also needed of dogs’ preferences, behaviors, and time budgets when provided access to multiple flooring surfaces in order to more comprehensively understand the implications for physical and behavioral health and hygiene.

In addition, selection bias due to voluntary participation by the kennels examined is likely to have occurred. However, even if such bias was present, the findings clearly suggest that it is possible to attain positive physical well-being outcomes in commercial breeding facilities using the flooring types investigated. The results also reiterate the importance of good management in influencing dog and kennel cleanliness and other positive welfare outcomes for breeding dogs maintained in commercial breeding operations.

## 5. Conclusions

This study’s findings indicate that diamond-shaped coated expanded metal, polypropylene, and concrete flooring types can all permit maintenance of dog cleanliness and foot health. Further, given that few serious elbow, hock, or paw problems were observed regardless of the flooring type, breed, or age of the dog, it appears that these aspects of dogs’ physical health can be maintained on these flooring types, particularly with appropriate management, which includes regular cleaning. The dogs’ ongoing access to additional surfaces via outdoor runs and exercise areas, as occurred at each of the facilities studied, may have provided additional foot health support (as has been reported for dairy cows with access to pasture [34]). Standardized cleaning protocols demonstrated to be effective in minimizing coliform recovery are needed to promote dog physical health and hygiene in commercial breeding kennels.

## Figures and Tables

**Table 1:** Dog information. Demographics of the population studied.

Facility	Breeds (n)	Sex		Age in Months (Mean ± SD, Range)
		Female (n)	Male (n)	
1	Yorkshire Terrier (10)	8	2	36.6 ± 18.6, 13–79
	Maltese (12)	11	1	
	Miniature Pinscher (8)	6	2	
2	Miniature Schnauzer (10)	6	4	31.6 ± 23.0, 12–118
	Havanese (10)	9	1	
	Coton de Tulear (10)	8	2	
3	Lhasa Apso (16)	14	2	47.1 ± 19.4, 25–106
	Min. Australian Shepherd (14)	13	1	
4	Rottweiler (8)	5	3	38.5 ± 12.7, 20–53
5	Weimaraner (7)	5	2	38.5 ± 12.7, 20–53
	Wheaton Terrier (7)	6	1	
	Dog de Bordeaux (1)	0	1	
	Pembroke Welsh Corgi (5)	4	1	

**Table 2:** Facility information. Types of indoor and outdoor flooring, size of dog housing, and amount of exercise at each facility. Note that facilities 4 and 5 each had indoor kennels with either DCEM or CON.

Facility	Indoor Housing			Outdoor Housing			Exercise Yard
	Type	Opening (cm)	Dimensions (m)	Type	Dimensions (m)	Access	Time/Day
1	DCEM	2.54 × 1.27	1.23 × 0.79	CON	1.23 × 0.79	04:30–17:00	1–2 h 3 days/week
2	POLY	2.54 × 2.54	1.42 × 1.12 or 1.42 × 0.94	CON	1.42 × 1.12 or 1.42 × 0.94	05:30–17:30	1 h 3 days/week
3	DCEM	3.81 × 1.91	1.52 × 0.91	CON	1.52 × 0.91	24 h/day	20 min 6 days/week
4	DCEM CON	2.54 × 1.27 N/A	1.25 × 1.22 1.52 × 1.22	CON	1.22 × 3.05 or 1.52 × 1.22	24 h/day	20–120 min daily
5	DCEM CON	3.18 × 1.91 N/A	1.22 × 1.22 or 1.22 × 0.91	CON	1.22 × 1.22 or 1.22 × 0.91	24 h/day	45–60 min 2 days/week

**Table 3:** Elbow, hock, and paw conditions. Name and description of conditions assessed.

Metric	Definition
Sore	A raw or painful place.
Wound	An injury to living tissue caused by a cut, blow, or other impact, typically one in which the skin is broken.
Nodule	A small swelling or aggregation of cells, especially an abnormal one.
Cyst	Closed capsule or sac-like structure, typically filled with fluid, semi-solid or gaseous material, similar to a blister.
Erythema	Superficial reddening of the skin, usually in patches, as a result of injury or irritation causing dilatation of the blood capillaries.
Alopecia	A loss of hair, partial or complete, in areas where it normally grows.
Callus	Thickened and hard part of the skin resulting from excess friction.
Abrasion	Scraped area on the skin or on a mucous membrane, resulting from injury or irritation.
Acral lick dermatitis (lick granuloma)	Lesion, typically on the distal portion of one or more limbs, resulting from excessive licking. May be red, swollen, irritated, bleeding, eventually becoming a thick, firm, plaque.
Pododermatitis	A condition characterized by inflammation of the dermal tissue of the paws.
Interdigital furuncles (cyst)	Painful nodular lesions located in the interdigital webs histologically representing areas of nodular pyogranulomatous inflammation.
Hyperkeratosis	Hypertrophy of the horny layer of the skin, or any disease characterized by it.
Hyperplasia	Abnormal increase in the volume of tissue caused by the formation and growth of new normal cells.
Matted	Hair or fur tangled into a thick mass.

**Table 4:** Physical exam. Summary of the results of the physical exam. BCS: body condition score.

Metric	Facility				
	1 (n = 30)	2 (n = 30)	3 (n = 30)	4 (n = 8)	5 (n = 20)
	Mean (SD)				
**BCS**	3.0 (0.7)	3.2 (0.5)	3.4 (0.6)	3.2 (0.4)	3.2 (0.5)
	Number of dogs with condition				
**Elbow/Hock**					
Alopecia	8	0	0	5	2
Calluses	1	0	0	0	2
Inflammation	0	0	0	0	1
**Paw**					
Hyperplasia	0	0	0	0	0
Hyperkeratosis	0	1	0	0	0
Matted	0	29	18	0	1
Erythema	0	3	3	1	0
Foot Pad Fissure	1	2	0	1	1
Cutaneous Lesion	4	1	0	0	1
Cyst	1	0	0	0	1
Alopecia	2	0	0	2	2
Inflammation	5	0	0	0	6
Dermatitis	1	0	0	0	1
Callus	0	0	1	1	0
Scar	1	1	0	0	0
	Mean (SD)				
**Toenail length (mean/dog)**	1.5 (0.5)	1.2 (0.3)	1.5 (0.5)	1.1 (0.2)	1.2 (0.4)

**Table 5:** Visual Body Cleanliness Scores (BCLS). Summary of the results of the cleanliness scoring by facility and floor type. DCEM: Diamond-shaped coated expanded metal; POLY: Polypropylene; and CON: Concrete.

Facility	Flooring	Observations	Mean	SD	Min.	Max.
1	DCEM	30	1.07	0.25	1	2
2	POLY	30	1.03	0.2	1	2
3	DCEM	30	1	0	1	1
4	DCEM	3	1	0	1	1
	CON	5	1.2	0.45	1	2
5	DCEM	10	1	0	1	1
	CON	10	1.04	0.2	1	2
Total	118	118	1.04	0.2	1	2

**Table 6:** Visual Kennel Cleanliness Scores. Summary of the results of the cleanliness scoring by facility and floor type. DCEM = Diamond-shaped coated expanded metal, POLY = Polypropylene and CON = Concrete.

Facility	Flooring	Observations	Mean	SD	Min.	Max.
1	DCEM	30	1	0	1	1
2	POLY	30	1.1	0.3	1	2
3	DCEM	27	1.5	0.5	1	2
4	DCEM	3	1	0	1	1
	CON	5	1	0	1	1
5	DCEM	10	1	0	1	1
	CON	10	1	0	1	1
Total		115	1.15	0.36	1	2

**Table 7:** Floor swabs. *E. coli* positive samples by facility and flooring type. DCEM: Diamond-shaped coated expanded metal; POLY: Polypropylene; and CON: Concrete.

Facility	Flooring	Samples	*E. coli* (+)	% of Samples
1	DCEM	29	9	31
2	POLY	30	8	19
3	DCEM	15	1	7
4	DCEM	9	2	22
	CON	11	0	0
5	DCEM	6	0	0
	CON	11	2	18

## Appendix B

**Table A1:** Cleanliness scoring tool. The tool used to score body and kennel cleanliness.

**Kennel Cleanliness Scores**
1- Acceptable: No debris is present. Dogs can rest comfortably. Food and water is not contaminated. 2- Marginal: No more than 1–25% of kennel is covered in debris. Dogs can easily avoid contact with debris. Slight odor is present. Food or drinking water shows no contamination. 3- Unsanitary: 26–50% of the kennel covered in debris. Debris may inhibit comfortable rest of dogs in kennels. A moderate odor is present. Food and water may be contaminated. 4- Very unsanitary: 51–75% of the kennel covered in debris inhibiting comfortable rest of dogs in kennel. Very difficult for dog to avoid debris. Food and water may be contaminated. 5- Filthy: >75% of the kennel covered in debris. Very difficult to impossible for dogs to avoid contact with debris. Food or drinking water is contaminated.
**Body Cleanliness Scores**
1- 0%: no debris present on the dog. 2- ≤25%: debris present on the paws only. 3- 26–50%: debris present on the legs, chest, and/or abdomen. 4- 51–75%: debris covering most of the dog except his/her head and neck. 5- 75%: debris present on all parts of the dog including the neck and head.
